# Supplementary material for: Certificateless broadcast signcryption scheme supporting equality test in smart grid
Source: PLoS One. 2023 Sep 7;18(9):e0290666. doi: 10.1371/journal.pone.0290666 (PMC10484466; doi:10.1371/journal.pone.0290666)
Supplement: Attachment — Submitted filename: Response to Reviewers.pdf [file pone.0290666.s001.pdf]

Original Manuscript ID: PONE-D-23-08826
Original Article Title: Certificateless broadcast signcryption scheme supporting equality test in smart grid

To: PLOS ONE Editor
Re: Response to reviewers

Dear Editor,

Thank you for allowing a resubmission of our manuscript, with an opportunity to address the reviewers' comments. Based on these comments, we have revised this paper carefully. All of the corrections made in the revision were marked in blue. We hope that it helps editor and reviewers finding the corrections.

We are uploading (a) our point-by-point response to the comments (below) (response to reviewers), (b) an updated manuscript with blue highlighting indicating changes, and (c) a clean, updated manuscript without highlights.

Best regards,

Shufen Niu, Runyuan Dong and Lizhi Fang

Comments of editor

Editor

Comments to the author

The paper has many serious issues. The conclusion section is missing, motivation is missing and the paper needs to be rewritten.

Author response: We sincerely thank you for the professional comment. We added a detailed motivation and conclusion for the proposed scheme.

Author action: In the revision, we updated the manuscript by meticulously proofreading and correcting these errors and mistakes, while also we added a detailed motivation (Page 2) and conclusion (Page 16) for the proposed scheme. Our modifications are as follows:

Motivation and contributions

To realize the private preservation of smart meter's identities and the confidentiality of sensitive information, while also saving bandwidth resources, we propose a broadcast signcryption scheme supporting equality test based on certificateless cryptosystem.The main contributions of our work as follows:

- The scheme ensures the privacy of the user's identity. Not only are illegal receivers unable to obtain the sender's identity but the receiver also do not know the other receiver's identity.
- The proposed scheme uses equality test to realize the function of data de-duplication. To achieve efficient utilization of network bandwidth, the duplicate ciphertext of the information generated by different encryption methods is de-duplicated by a trusted third party.
- We realize lightweight broadcast signcryption by reducing the bilinear pairing operation with a high computation cost in unsigncryption. The experimental analysis showed that the computing efficiency was higher than existing schemes, and had greater advantages in practical applications.

Conclusion

Currently, there exist malicious attackers in the smart grid, causing the smart grid to face some security threats, such as user forging smart meter data, unauthorized user access to sensitive information leading to privacy leakage, and so on. To realize the private preservation of smart meter's identities and the confidentiality of sensitive information, guarantee the security of data communication and solve the problem of insufficient transmission network bandwidth resources, we construct a broadcast signcryption scheme supporting equality test based on certificateless cryptosystem.The scheme realizes the anonymity between receivers and ensures the privacy of data. In addition, our work also achieves data deletion function of the same ciphertext, which greatly saves the network bandwidth and ciphertext storage space. Finally, an analysis of the existing broadcast signcryption schemes and our proposed scheme reveals that our proposed work has higher practical application value.

Comments of reviewers

Reviewers:1

Comments to the author

The authors seek to propose a broadcast signcryption scheme supporting equality test based on certificateless Cryptosystem for smart grid. I have the following suggestions that need to be answered. emerging from their work they should address:

Reviewer#1, Conceren#1:

In the abstract, the sentence needs to be revised. "However, the current scheme cannot ensure user privacy or efficient network bandwidth utilization."

Author response: We are very grateful for this suggestion. According to your suggestion, we revised the sentence "However, the current scheme cannot ensure user privacy or efficient network bandwidth utilization."

Author action: In the revision, we revised the sentence "However, the current scheme cannot ensure user privacy or efficient network bandwidth utilization." to "However, most of the existing schemes require a large amount of network bandwidth resources and cannot ensure the receiver's anonymity."

Reviewer#1, Conceren#2:

I would suggest the authors to add a detailed motivation for the proposed scheme.

Author response: We sincerely thank you for the professional comment. We added a detailed motivation for the proposed scheme.

Author action: In the revision, we added a detailed motivation for the proposed scheme in motivation and contributions (Page 2). Our modifications are as follows:

Motivation and contributions

To realize the private preservation of smart meter's identities and the confidentiality of sensitive information, while also saving bandwidth resources, we propose a broadcast signcryption scheme supporting equality test based on certificateless cryptosystem.The main contributions of our work as follows:

- The scheme ensures the privacy of the user's identity. Not only are illegal receivers unable to obtain the sender's identity but the receiver also do not know the other receiver's identity.
- The proposed scheme uses equality test to realize the function of data de-duplication. To achieve efficient utilization of network bandwidth, the duplicate ciphertext of the information generated by different encryption methods is de-duplicated by a trusted third party.
- We realize lightweight broadcast signcryption by reducing the bilinear pairing operation with a high computation cost in unsigncryption. The experimental analysis showed that the computing efficiency was higher than existing schemes, and had greater advantages in practical applications.

Reviewer#1, Conceren#3:

A detailed paragraph regarding the hardness efficiency details of the proposed scheme

needs to be added above the contribution's headings. The authors need to show why the proposed scheme is efficient and how.

Author response: We are extremely grateful for your comment. We added the hardness efficiency details of the proposed scheme to show why the proposed scheme is efficient and how in the introduction.

Author action: In the revision, we added the hardness efficiency details of the proposed scheme to show why the proposed scheme is efficient and how in Section 1 (Page 2). Our modifications are as follows:

Currently, there exist malicious attackers in the smart grid, causing the smart grid to face some security threats, such as user forging smart meter data, unauthorized user access to sensitive information leading to privacy leakage, and malicious attacker stealing data when wireless sensor networks transmit information over public channels. Thus, the scheme demands the intelligent power supply system encrypt data and send ciphertext to users in the form of broadcasting, so as to transmit users' power information efficiently and securely. Broadcast signcryption achieves data sharing between broadcast servers and authorized receivers. Unfortunately, many existing broadcast signcryption schemes have some shortcomings. They are unable to realize the private preservation of receiver identities and need a lot of bandwidth resources.

Based on the current security threat in smart grid and the shortcomings of existing schemes, we propose a broadcast signcryption scheme that supports equality test based on certificateless cryptosystem. First, the scheme solves the key escrow problem in identity-based cryptosystem by using certificateless cryptosystem and ensures the receiver's anonymity by using the Lagrange interpolation theorem. Second, our proposed scheme can be proven secure under the Random Oracle Model. In addition, the proposed scheme realizes the function of data de-duplication by using equality test and lightweight broadcast signcryption by reducing computation cost.

Reviewer#1, Conceren#4:

The entire literature review section is written in the present tense. The words like constructed, presented, and proposed need to be changed to propose, construct and present.

Author response: We are very grateful for this suggestion. According to your valuable suggestion, we have carefully revised our manuscript. We changed words like constructed, presented and proposed using the present tense.

Author action: We changed words like constructed, presented and proposed using the present tense (Page 2). Our modifications are as follows:

Duan et al. [12] first construct the broadcast signcryption scheme in combination with the signcryption algorithm and the broadcast transmission.Unfortunately, the scheme does not meet the security requirements of adaptive chosen-ciphertext attack. Based on the problem of one-to-one single transmission in the traditional signcryption scheme, broadcast signcryption solves the shortcomings of communication efficiency in information transmission. Since the invention of broadcast signcryption, many academics and practitioners have propose the scheme to meet various security performance requirements [13, 14]. Zhang et al. [13] construct a signcryption scheme that resists quantum attacks based on lattice and identity cryptosystem. [14,15] designed the efficient signcryption algorithm that allowed the sender to transmit multi-messages to multi-receivers and analyzed the efficiency of each scheme. Qiu et al. [15] design a broadcast

---

Reviewer#1, Conceren#5:

The related work is limited; I would suggest the authors expand the related work. Besides,

I would suggest the authors to conclude the related work. Currently, the authors did not conclude the related work; what did they learn after reviewing the literature?

Author response: Thank you very much for your invaluable suggestion. We expanded the related works and added the conclusion of related works in Section 2.

Author action: In the revision, we expanded the related works and added the conclusion of related works in Section 2 (Page 3). Our modifications are as follows:

Related works

Duan et al. [12] first construct the broadcast signcryption scheme in combination with the signcryption algorithm and the broadcast transmission.Unfortunately, the scheme does not meet the security requirements of adaptive chosen-ciphertext attack. Based on the problem of one-to-one single transmission in the traditional signcryption scheme, broadcast signcryption solves the shortcomings of communication efficiency in information transmission. Since the invention of broadcast signcryption, many academics and practitioners have propose the scheme to meet various security performance requirements [13, 14]. Zhang et al. [13] construct a signcryption scheme that resists quantum attacks based on lattice and identity cryptosystem. [14,15] designed the efficient signcryption algorithm that allowed the sender to transmit multi-messages to multi-receivers and analyzed the efficiency of each scheme. Qiu et al. [15] design a broadcast scheme based on certificateless cryptosystem and applied it to the IoT, lowering the computation cost of the receiver by outsourcing the gateway signature verification operation.However, there is the problem of key escrow. Peng et al. [16] connected the edge node with the IoT device. Edge computing can reduce the computation burden of terminal devices and the delay of network transmission. However, the ciphertext of this scheme hasn't the authorization set of the receiver. Due to the risk of location privacy leakage in the charging process of electric vehicles. Kumar et al. [17] design an electric vehicle charging framework combined with grid encryption technology. Alagarsamy et al. [18] propose an Exponentiated Multilinear Vectorized Certificateless Signcryption (EMV-CLSC) scheme, which reduces memory usage when processing multiple data and improves computation efficiency. [19–21] propose lightweight and efficient access control signcryption schemes based on the certificateless cryptosystem. Ullah et al. [20] propose an anonymous certificateless signcryption scheme using elliptic curves to guarantee security requirements in Internet of Vehicles, but this scheme only signcrypt single message and is not suitable for the multi-message environments. Sarvesh et al. [22] present a multi-signcryption scheme with public verifiability to reduce the threats of private key escrow and replay attacks. Unfortunately, These schemes fail to consider the processing of redundant data generated by different encryption methods for the same information. Luo et al. [23] propose the signcryption scheme for data communication between different network domains, but can't ensure the privacy of receivers. Khan et al. [24] set a smaller key unit based-identity signcryption, which is not applicable to equipment with limited resources, and there is the risk of the receiver's privacy leaking. Mandal et al. [25] design a user access control scheme that fails to achieve the receiver's privacy preservation. Shen et al. [26] propose a lightweight and secure data transmission protocol for wireless body area networks, which support the multidisciplinary treatment but exist a risk of leakage of the partial private key.

Aiming at addressing the shortcomings and improving the efficiency of existing schemes, we propose a broadcast signcryption scheme that supports equality test based on certificateless cryptosystem. Our proposed work ensures the receiver's anonymity and information integrity and confidentiality, while also the proposed scheme realizes the function of data de-duplication by using equality test and lightweight broadcast signcryption by reducing computation cost.

Reviewer#1, Conceren#6:

In the system model, the authors just define the terminology used in the scheme; however, the workflow of the proposed scheme is missing.

Author response: We are very grateful for this suggestion. According to your valuable suggestion, we have carefully revised our manuscript. We added the workflow of the proposed scheme to the system model.

Author action: In the revision, we added the workflow of the proposed scheme in the system model (Page 4). Our modifications are as follows:

As is shown in Fig 2, our proposed system model includes five entities: Intelligent power supply system, Trusted Third Party (TTP), Key Generation Center (KGC), Smart meter and Cloud Server (CS). After the KGC generates public and private key for the intelligent power supply system and the smart meter, the intelligent power supply system will signcrypt the collected information such as the meter operation status and smart meter data, and then sends ciphertext to CS and TTP for ciphertext equality test. TTP deletes the duplicate ciphertext of the information generated by different encryption methods. When the CS broadcasts ciphertext to smart meter, the authorized device can unsigncrypt ciphertext using private key independently.

Reviewer#1, Conceren#7:

The scheme's definition needs to be named with the subsection, like System Definition.

Author response: We sincerely thank you for the professional suggestion and comment. We remove the scheme's definition from the system model subsection and create a subsection for our scheme' definition.

Author action: We remove the scheme's definition from the system model subsection and create a subsection for our scheme's definition (Page 5). Our modifications are as follows:

Formal definition

For the signcryption scheme supporting equality test in smart grid, we give the detailed definition as follows for algorithm:

Setup (1^λ)**:** Inputs security parameter λ, KGC returning public parameter $Pars$, TTP's private key x_T and the master key s.

Set secret-value (ID_i)**:** It takes $Pars$ and the receiver's identity ID_i as input and returns x_i as the receiver's secret value.

Extract partial-private key (s, X_i, ID_i)**:** The inputs are the ID_i of the receiver, the master key s, the public key of the receiver X_i and the public parameters $Pars$, and it returns the partial private key z_i.

Reviewer#1, Conceren#8:

I haven't seen any details in the experimental analysis for the number of devices. How and from where did the authors add the details for the number of devices? How can the authors set the devices hypothetically? The authors did not conclude their research; a conclusion is necessary and must be added.

Author response: We sincerely thank you for the professional suggestion and comment. We simulate the experiment using bilinear pairing-based cryptography library under the Linux operating system. So the number of devices is manually set according to the existing scheme settings. In the experiment, the number of devices can also be used to indicate the number of smart meters in the smart grid. The number of devices on the smart meter can be dynamically adjusted to manage authorized devices more flexibly. Based on the reviewer's suggestion, we modified the number of devices to the number of smart meters in Fig 3, Fig 4, Fig 5 and added the conclusion to the manuscript.

Author action: In the revision, we modified the number of devices to the number of smart

meters in Fig 3, Fig 4, Fig 5 and added the conclusion of our research to the manuscript in Section 7 (Page 16). Our modifications are as follows:

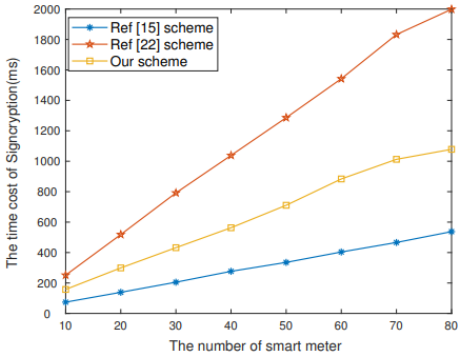

Fig 3. Time cost of signcryption

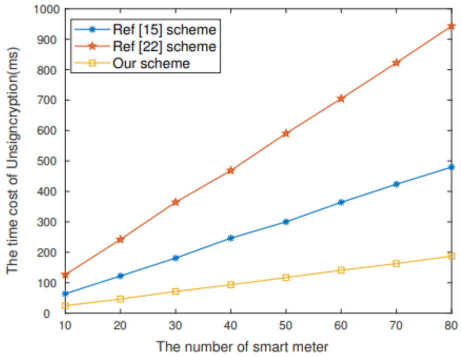

Fig 4. Time cost of unsigncryption

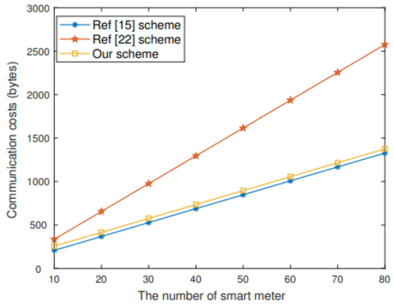

Fig 5. Communication costs

Conclusion

Currently, there exist malicious attackers in the smart grid, causing the smart grid to face some security threats, such as user forging smart meter data, unauthorized user access to sensitive information leading to privacy leakage, and so on. To realize the private preservation of smart meter's identities and the confidentiality of sensitive information, guarantee the security of data communication and solve the problem of insufficient transmission network bandwidth resources, we construct a broadcast signcryption scheme supporting equality test based on certificateless cryptosystem.The scheme realizes the anonymity between receivers and ensures the privacy of data. In addition, our work also achieves data deletion function of the same ciphertext, which greatly saves the network bandwidth and ciphertext storage space. Finally, an analysis of the existing broadcast signcryption schemes and our proposed scheme reveals that our proposed work has higher practical application value.

Reviewer#1, Conceren#9:

The article needs to be thoroughly proofread to remove all the grammatical and typos.

Author response: We apologize for the language problems in the original manuscript. According to the reviewer's suggestions, we have corrected the errors and mistakes with due diligence and asked a native English speaker to edit and enhance the standard of English of our manuscript.

Author action: We updated the manuscript by meticulously proofreading and correcting these errors and mistakes. This revision is highlighted in blue in the text.

Reviewers:2

Comments to the author

The technical aspects of the paper are fine. The authors, however, will need to make some minor adjustments before publishing it. The following are some of the concerns that the authors should address:

Reviewer#2, Conceren#1:

The introduction is quite brief. The authors should add to it, highlighting the smart grid's vulnerabilities and the merits of using a certificateless signcryption scheme.

Author response: We are very grateful for this suggestion. According to your valuable suggestion, we have carefully revised our manuscript. We added the smart grid's vulnerabilities and the merits of using a certificateless signcryption scheme in introduction.

Author action: In the revision, we added the smart grid's vulnerabilities and the merits of using a certificateless signcryption scheme in the introduction (Page 2). Our modifications are as follows:

Currently, there exist malicious attackers in the smart grid, causing the smart grid to face some security threats, such as user forge smart meter data, unauthorized user access sensitive information leads to privacy leakage and malicious attacker steal data when wireless sensor networks transmit information over public channels. Thus, the scheme demands the intelligent power supply system encrypt data and send ciphertext to users in the form of broadcasting, so as to transmit users' power information efficiently and securely. Broadcast signcryption achieves data sharing between broadcast servers and authorized receivers. Unfortunately, many existing broadcast signcryption schemes have some shortcomings. They are unable to realize the private preservation of receiver identities and need a lot of bandwidth resources.

Based on the current security thereat in smart grid and the shortcomings of existing schemes, we proposed a broadcast signcryption scheme that supports equality test based on certificateless cryptosystem. First, the scheme solves the key escrow problem in identity-based cryptosystem by using certificateless cryptosystem and ensures the receiver's anonymity by using the Lagrange interpolation theorem. Second, our proposed scheme can be proven secure under the Random Oracle Model. In addition, the proposed scheme realizes the function of data de-duplication by using equality test and lightweight broadcast signcryption by reducing computation cost.

Reviewer#2, Conceren#2:

Once more, the literature review section is extremely brief. Few relevant articles precisely on the same subject are ignored, for example (https://doi.org/10.3390/su131910891). The authors must contribute a few additional articles to this section.

Author response: We are very grateful for this suggestion. According to your valuable suggestion, we added the literature review in Section 2.

Author action: In the revision, we added the relevant articles precisely on the same subject in Section 2 (Page 3). Our modifications are as follows:

improves computation efficiency. [19–21] propose lightweight and efficient access control signcryption schemes based on the certificateless cryptosystem. Ullah et al. [20] propose an anonymous certificateless signcryption scheme using elliptic curves to guarantee security requirements in Internet of Vehicles, but this scheme only signcrypt single message and is not suitable for the multi-message environments. Sarvesh et al. [22] present a multi-signcryption scheme with public verifiability to reduce the threats of private key escrow and replay attacks. Unfortunately, These schemes fail to consider the processing of redundant data generated by different encryption methods for the same information. Luo et al. [23] propose the signcryption scheme for data communication between different network domains, but can't ensure the privacy of receivers. Khan et al. [24] set a smaller key unit based-identity signcryption, which is not applicable to equipment with limited resources, and there is the risk of the receiver's privacy leaking. Mandal et al. [25] design a user access control scheme that fails to achieve the receiver's privacy preservation. Shen et al. [26] propose a lightweight and secure data transmission protocol for wireless body area networks, which support the multidisciplinary treatment but exist a risk of leakage of the partial private key.

Aiming at addressing the shortcomings and improving the efficiency of existing schemes, we propose a broadcast signcryption scheme that supports equality test based on certificateless cryptosystem. Our proposed work ensures the receiver's anonymity and information integrity and confidentiality, while also the proposed scheme realizes the function of data de-duplication by using equality test and lightweight broadcast signcryption by reducing computation cost.

Reviewer#2, Concern#3:

I could not locate the section's conclusion. I have no idea why the conclusion is missing. Authors must provide justification.

Author response: We sincerely thank you for the professional comment. According to your suggestion, we added the conclusion to the manuscript.

Author action: In the revision, we added the conclusion in Section 7 (Page 16). Our modifications are as follows:

Conclusion

Currently, there exist malicious attackers in the smart grid, causing the smart grid to face some security threats, such as user forging smart meter data, unauthorized user access to sensitive information leading to privacy leakage, and so on. To realize the private preservation of smart meter's identities and the confidentiality of sensitive information, guarantee the security of data communication and solve the problem of insufficient transmission network bandwidth resources, we construct a broadcast signcryption scheme supporting equality test based on certificateless cryptosystem.The scheme realizes the anonymity between receivers and ensures the privacy of data. In addition, our work also achieves data deletion function of the same ciphertext, which greatly saves the network bandwidth and ciphertext storage space. Finally, an analysis of the existing broadcast signcryption schemes and our proposed scheme reveals that our proposed work has higher practical application value.

Reviewer#2, Concern#4:

The authors need to revise the article with correct usage of English, grammatical mistakes, and punctuation errors.

Author response: We apologize for the language problems in the original manuscript. According to the reviewer's suggestions, we have corrected the errors and mistakes with due diligence and asked a native English speaker to edit and enhance the standard of English of our manuscript.

Author action: We updated the manuscript by meticulously proofreading and correcting these errors and mistakes. This revision is highlighted in blue in the text.

We are very grateful for your valuable comments. We carefully revised the manuscript following the Reviewers' suggestions, tried our best to improve the manuscript, and made some changes in manuscript. These changes will not influence the content and framework of the paper. We appreciate Editors/Reviewers' warm work earnestly and hope that the correction will meet with approval. Once again, thank you very much for your comments and suggestions.
